# Barriers and enablers to Caregivers Responsive feeding Behaviour (CRiB): A mixed method systematic review protocol

**DOI:** 10.12688/hrbopenres.12980.2

**Published:** 2020-06-10

**Authors:** Vicki Slater, Jennie Rose, Ellinor Olander, Karen Matvienko-Sikar, Sarah Redsell

**Affiliations:** 1 Faculty of Health, Education, Medicine and Social Care, Anglia Ruskin University, Cambridge, CB1 1PT, UK; 2 Centre for Maternal and Child Health Research, City, University of London, London, EC1V 0HB, UK; 3 School of Public Health, University College Cork, Cork, T12 HY8E, Ireland

**Keywords:** Responsive feeding, caregiver, infant, overweight, obesity, systematic review, protocol

## Abstract

**Background**: Childhood overweight and obesity is a major public health issue. Responsive feeding has been identified as having a protective effect against child overweight and obesity, and is associated with healthy weight gain during infancy. Responsive feeding occurs when the caregiver recognises and responds in a timely and developmentally appropriate manner to infant hunger and satiety cues. Despite its benefits, responsive feeding is not ubiquitous. To better support caregivers to engage in responsive feeding behaviours, it is necessary to first systematically identify the barriers and enablers associated with this behaviour. This mixed-methods systematic review therefore aims to synthesise evidence on barriers and enablers to responsive feeding using the COM-B model of behavioural change.

**Methods**: 7 electronic databases will be searched (Maternal and Infant Care, CINAHL, Cochrane, PubMed, Medline, PsycINFO, EMBASE). Studies examining factors associated with parental responsive and non-responsive feeding of infants and children (<2 years) will be included. Papers collecting primary data, or analysing primary data through secondary analysis will be included. All titles, abstracts and full texts will be screened by two reviewers. Quantitative and qualitative data from all eligible papers will be independently extracted by at least two reviewers using pre-determined standardised data extraction forms. Two reviewers will independently assess the methodological quality of the studies using the Mixed Methods Appraisal Tool (MMAT). This review will be reported according to the Preferred Reporting Items for Systematic reviews and Meta Analyses (PRISMA).

**Ethics and dissemination**: Ethical approval is not required for this review as no primary data will be collected, and no identifying personal information will be present. The review will be disseminated in a peer reviewed journal.

**PROSPERO registration**: CRD42019144570 (06/08/2019)

## Introduction

The number of children overweight under the age of five is estimated to be over 41 million ^1^, leading to prevalence of overweight and obesity in infants and children being identified as a major public health issue ^2^. Infancy is posited to be a sensitive period for the development of child overweight, particularly the first two years ^3, 4^. The first 2 years are especially important because, during this time, children's feeding preferences and behaviours are influenced by modifiable parental approaches to child feeding that can lead to childhood obesity ^5, 6^. Throughout this time, infants experience bottle and breastfeeding, complementary feeding ^7^, leading onto a solid diet. Beyond this, other critical aspects of child development occur, including development of attachment ^8^ and self-regulation ^9^, and attention ^10^ which may interact with feeding. For instance, during the first 2 years of life, infants learn to self-regulate their food intake, from their own appetitive traits ^11^ and from their environment ^12^. Childhood obesity can lead to immediate and long term health complications, including, obstructive sleep apnoea, high blood pressure and obesity related cardiovascular disease ^13^. Children who have obesity are more likely to have obesity in adulthood, which is associated with a higher risk of many chronic diseases ^14^. Parental feeding practices and styles (as outlined in Table 1) are a crucial determinant in the aetiology of childhood obesity ^15^, with responsive feeding (both bottle and breast feeding) identified as having a protective effect against child overweight and obesity, and an associated reduced risk of overweight and obesity ^16, 17^.

**Table 1.** Table demonstrating definitions of different parental feeding styles and how they may relate to childhood overweight.

Parental feeding styles example	Definition
Instrumental feeding	Using food as a reward for a desired outcome (i.e. a positive behaviour). This may strengthen the preference for that food (often high calorie) ^19^ .
Pressuring to eat	Prompting to eat more food; the caregiver is concerned with increasing the child’s food intake (such as adding cereal to a child’s bottle to increase intake) ^22^.
Monitoring food intake	Monitoring a child’s food intake; may be expected to result in a lower BMI, however research has often identified no weight change ^23^.
Responsive feeding	Responding promptly and in a developmentally appropriate manner to infant cues of satiety and hunger ^3^.
Food restriction	Minimising access to food to reduce child’s weight. This can result in the opposite effect by causing the child to seek out the restricted food ^19^.

Responsiveness is a reciprocal dimension of feeding in which an infant or young child provides clear feeding cues, such as hunger and satiety, and the caregiver responds in a prompt and developmentally appropriate manner ^3^. Responsive feeding can relate to early consumption of breast and/or formula milk, as well as in relation to introducing and establishing solid food consumption. From a very young age infants have the ability to self-regulate their food intake ^18^ but the volume of food an infant consumes depends on their caregiver’s ability to recognise and respond appropriately to their infant’s hunger and satiety cues, as well as this ability to self-regulate their intake. Non-responsive feeding may occur between an infant and caregiver when a caregiver misinterprets or misunderstands the infant’s hunger and satiety cues, and so responds by offering a developmentally inappropriate amount, type or texture of food. Non-responsive feeding may include, instrumental feeding, pressuring a child to eat, and controlling food intake, which have all been associated with childhood overweight and obesity ^19– 21^. Non-responsive feeding can be conceptualised as being at the opposite end of the spectrum to responsive feeding and research suggests it has a role in childhood weight gain and overweight ^3^. For example, caregivers who have an inability to recognise an infant’s weight is within a healthy range (with the infant being underweight or overweight), often utilise non-responsive feeding styles such as food restriction (for those infants perceived as overweight), or pressuring-to eat (for those infants perceived as underweight) ^24, 25^. These feeding styles have been associated with children (from birth through to age 18 years inclusive) developing unhealthy eating styles (such as emotional eating and eating in the absence of hunger), leading to an increased risk of obesity ^26– 28^.

Multiple factors may affect how caregivers engage in feeding behaviours. A recent qualitative review included studies consisting of predominantly or only mothers, in Europe, North America, Australasia and Mexico, with the majority of studies recruiting low-income caregivers ^29^. This review explored parental experiences of infant feeding and highlighted that some of these influencing factors are environmentally based.). These included costs of infant feeding ^30^, time constraints ^31^, sources of information (official recommendations ^32^, healthcare professionals ^33^, and friends and family ^30^, confusion of information from multiple sources ^34^, and pressure from family ^35^. Additional factors include psychological factors (such as maternal mental health ^36^ and maternal executive functioning ^37^), and social factors (including, interpersonal relationships, marital status, occupation, and the influence of family and friends) ^29^. Parental knowledge, beliefs, and prior experience also influence their feeding practices and styles ^29^. Previous literature, such as an analysis of the GEMINI cohort study, has noted the importance of genetics in appetitive traits including slowness in eating, satiety responsiveness, food responsiveness and enjoyment of food ^11^. Caregivers may also be simply responding to their infant’s appetite ^12^ with infants varying in appetite ^38^. Additionally, parents have been found to adjust their feeding style dependent on their child’s weight; an infant who is perceived by the parent to be underweight may be pressured to eat more, whilst parents may restrict their infant’s food intake if they perceive their infant to be overweight ^24^. However, evidence is mixed for maternal pressure to eat, with some research suggesting pressure to eat is associated with increased risk of obesity ^3^, meanwhile it has also been reported that pressure to eat is associated with lower weight at 2 years ^21^. Infant temperament may also influence feeding practices, with infants perceived as fussier, less responsive to food, and more responsive to internal satiety cues being pressured more ^39^. Although the evidence is mixed, it appears that responsive feeding is protective against the development of childhood obesity ^40^.

Although sources such as the WHO UNICEF Baby Friendly Initiative (BFI) exist to provide guidance on improving caregivers’ knowledge about responsive feeding ^41, 42^, parents still report uncertainty about how ‘best’ to feed their infants ^29^. It is also suggested that healthcare professionals have not been equipped appropriately to assist caregivers in responsive feeding ^43^. In order to improve information and support for caregivers it is necessary to examine the barriers and enablers to responsive feeding. Understanding the underlying factors that influence responsive feeding will contribute to the development of a caregiver-focused support that aids responsive feeding. Systematic reviews have reported that healthcare professionals providing responsive feeding guidance to mothers on identifying and responding to children’s satiety and hunger cues can lead to healthy weight status/gain in early childhood ^18, 44– 47^.

Of central importance to improving and supporting appropriate responsive feeding behaviours is the fact that some determinants of this behaviour are modifiable, such as caregiver knowledge, and may be specifically targeted through interventions. Models of behaviour change are fundamental to informing such interventions and strategies to promote positive public health ^48^. The COM-B (capability, opportunity, motivation and behaviour) model for example provides a framework for understanding behaviour change, and incorporates ‘capability’, ‘opportunity’, and ‘motivation’ are conceptualised as the three conditions necessary for behavioural change ^48^. Utilising the COM-B model to map barriers and enablers of responsive feeding behaviours provides a useful and tangible first step towards development of interventions and supports to assist primary caregivers to engage in responsive feeding behaviours that are associated with reduced risk of childhood obesity.

### Research questions

What are the barriers and enablers associated to responsive and non-responsive feeding to prevent childhood overweight and obesity?

## Method

### Study registration

This study has been registered with the international Prospective Register of Systematic Reviews on 6 ^th^ August 2019 (PROSPERO; registration number, CRD42019144570).

### Study design

A step-by-step flow diagram will be used in accordance with the Preferred Reporting Items for Systematic review and Meta-analysis protocol (PRISMA-P) guidelines, to demonstrate the study selection process, and rationale will be provided for excluded studies. The entirety of the review will follow the PRISMA-P checklist.

### Ethics

Ethical approval is not required for this review as no experimental or observational research will be carried out, and no identifying personal information will be present or collected.

### Types of studies

This review will examine both qualitative and quantitative primary studies that have examined factors associated with caregiver responsive and nonresponsive feeding of children up to 2 years old. All studies collecting primary data, or analysing primary data through secondary analyse will be included. Quantitative research such as, randomised control trials, case-control studies, retrospective and prospective cohort studies, cross-sectional and longitudinal studies will be included. In addition, qualitative studies, including research conducted as part of the process evaluation of an intervention trial, will be included. A broad remit of studies will be included in order to ensure factors that emerge in a variety of contexts and settings are identified. The studies must be published in English due to limitations in translation resources, and there will be no restriction on publication date.

## Inclusion and exclusion criteria

### Population

Primary caregivers (parents, guardians) of healthy children ≤ 2 years old. Studies of infants with medical conditions affecting feeding and growth, very preterm infants <32 weeks gestation, low birth weight (VLBW) <2500 g ^49^, and those who have been fed via a naso-gastric tube will be excluded from this review. We will also exclude studies including infants with major sensory and physical disabilities (e.g. blindness, deafness) because of the additional challenges that caregivers of these infants may find implementing responsive feeding in early life. To ensure the findings can contribute to the development of an intervention to reduce the risk of childhood overweight in a UK and Ireland-relevant population, studies conducted in countries where responsive feeding is used to improve weight gain in malnourished infants will be excluded. Studies will only be included if they are carried out in an economically developed country (as indicated by membership of the Organisation for Economic Co-operation and Development (OECD)) ^50^.

### Exposures

The exposures of interest are the barriers and enablers associated with primary caregiver feeding responsiveness and non-responsiveness. Examples of non-responsive feeding include, pressuring a child to eat, instrumental feeding, and controlling food intake which have all been associated with childhood overweight and obesity ^19– 21^.

### Outcomes

To be included, studies need to report a factor that could be a barrier or enabler to responsive feeding, for example an intervention that includes anticipatory guidance. Responsive feeding during first 2 years of life as reported by the study authors. This will include outcomes measured using established scales, e.g. Child Feeding Questionnaire ^51^, and qualitative data in relation to caregiver feeding practices (such as, ensuring feeding context with few distractions) ^52^. Results from quantitative studies (for example, p-values, odds ratios, and confidence intervals) will be used to determine the existence and strength of associations between factors and feeding, whilst results from the qualitative studies (such as themes) will be synthesised to narratively explore barriers and enablers experienced by caregivers to responsive feeding.

## Method for identifying studies for inclusion

The following databases will be searched: CINAHL, Cochrane Library, Medline, Embase, PubMed, PsycINFO, Maternity and Infant Care database. All databases will be searched from inception. All databases will be searched using the comprehensive search strategy outlined below.

### Search strategy

The searches will be based on concepts associated with infant feeding behaviours to include proxy terms for responsive and non-responsive feeding and any barriers or enablers to primary caregiver engagement. We will use the following search strategy:

*Feeding type concept:* authoritarian OR authoritative OR bottle feeding OR breastfeeding OR breast feeding OR breast-feeding OR complementary feeding OR controlled feeding OR controlling feeding OR emotional feeding OR formula feeding OR non-responsive* OR pressured OR restricted feeding OR restricting feeding OR responsive* OR self-feeding OR unresponsive* OR weaning

*Influencing factors concept:* barrier* OR belief* OR challenge* OR determinant* OR enabler* OR experiences OR facilitator* OR facto* OR influenc* OR obstacle* OR parenting style* OR risk OR risk factors OR view*

*Subject concept:* babies OR baby OR child OR infant* OR maternal OR mother* OR neonat* OR newborn* OR parent* OR paediatric OR pediatric OR toddler*

*Study design concept:* cohort OR cross-sectional OR experiment* OR intervention OR interview OR observation* OR process evaluation OR qualitative.

### Study selection

One researcher (VS) will independently screen titles and abstracts of all identified papers against eligibility criteria. Two other researchers (JR, SR) will each screen titles and abstracts of half of the identified papers. At least two members of the researcher team (VS, JR, KM, EO, SR) will then independently screen full texts of potentially eligible articles for inclusion. Any discrepancies will be resolved by discussion or recourse to a third reviewer from the team (VS, JR, KM, EO, SR). If necessary, the reviewers will attempt to contact authors of original articles to request missing information or for clarification. All references will be imported into EndNote and duplicates will be removed through EndNote and through manual screening.

### Data extraction

Raw data from qualitative studies will be extracted onto an Excel spreadsheet and qualitative and quantitative data will be extracted using pre-determined standardised data extraction forms (see extended data ^53, 54^).

For the qualitative data extraction one researcher (SR) will extract the study participant, setting and design details of each paper and another researcher (JR) will download any qualitative data from each study to word files. Qualitative data will include the quotes, interpretative text and any other supplementary data. Two researchers (JR, SR) will each examine the qualitative data from three of the included papers and code the data relevant to barriers and enablers to responsive feeding to the COM-B framework. The researchers will meet to compare their interpretation of the data and coding, and any discrepancies will be discussed and resolved.

The quantitative data will be extracted independently by two reviewers (KM, EO), with one researcher (VS) extracting data from all quantitative studies, whilst two more researchers (KM, EO) will each extract data from half of the identified studies. The general study details (including author, title, date) will be extracted along with more specific details such as participant information, infant weight, and intervention details. Results of the study will be recorded (such as, confidence intervals, p-values, and standard deviations). Identified determinants and association factors identified in quantitative studies will be mapped onto the COM-B model, and will be synthesised with consideration given to the context of the strength of associations and effects. Researchers (VS, KM, EO) will meet to discuss findings of the data extraction and resolve any discrepancies.

### Assessment of risk of bias

Two reviewers (VS, SR) will independently assess the methodological quality of these studies using the Mixed Methods Appraisal Tool (MMAT) ^55^; any discrepancies will be resolved through consensus discussion or recourse to a third member of the research team (JR, KM, EO). MMAT provides two screening questionnaires, which are used in the appraisal stage of mixed methods systematic reviews. The MMAT is used to appraise five study types: randomised control trials, non-randomised studies, quantitative studies, qualitative research, and mixed methods design studies.

### Strategy for data synthesis

We will use narrative text along with tables of the findings from the included studies, structured around: 1) the relation of barriers and enablers to responsive feeding and non-responsive feeding, and 2) the existence and strength of association between factors and responsive and/or non-responsive feeding outcomes. Depending on the heterogeneity of quantitative studies identified, a meta-analysis will be conducted.

To synthesise the extracted qualitative data, we will use a ‘best fit’ framework synthesis, as outlined by Booth and Carroll ^56^. Framework synthesis is a structured approach in which data are analysed using concepts or themes specified *a priori* ^57, 58^. The ‘best fit’ approach follows seven distinct steps, which includes incorporation of inductively emerging themes with pre-specified themes within the *a priori* framework. This allows for a flexible and rigorous approach to qualitative evidence synthesis ^59^. It provides a pragmatic approach to providing context-specific information and understanding of parents’ experiences of, and barriers and facilitators to responsive feeding. The framework to be used is the Capability, Opportunity, and Motivation Model of Behaviour (COM-B model) ^48^, and findings will be mapped onto this model.

Participant quotations and authors’ interpretations in the results sections of included papers will be coded using the *a priori* COM-B framework. An inductive thematic analysis of the data will also be conducted and additional themes, which are not accounted for by the COM-B model, will be added to the coding framework. Concepts from the COM-B framework and inductive thematic analysis will then be revisited and synthesised into a final set of themes.

Quantitative data will be extracted onto the COM-B model, with evidence of each barrier and enabler to responsive feeding. All stages of analysis will be conducted by one researcher (VS) and will be reviewed and discussed by all members of the study team to reach consensus on the final evidence synthesis.

### Subgroup/subset analysis

Subgroup analysis will be determined and led by the data, but may include high/low income, mothers/fathers, primi/multiparous mothers.

### Dissemination of findings

The results of this systematic review will be published in a peer-reviewed journal.

### Study status

As of the 6 ^th^ January 2020, the selected databases have been searched, titles/abstracts have been screened, full texts have been screened against eligibility criteria, and data extraction has started.

## Discussion

The aim of this systematic review is to analyse the scientific literature exploring and reporting on barriers and enablers to responsive feeding. The findings will inform researchers, health professionals and caregivers about the ways in which responsive feeding during infancy might be promoted, supported and improved. This could include identification of the groups of caregivers who find responsive feeding more challenging and a clear understanding of the behavioural components which may make this difficult. This should inform the co-production of specific education and support packages for both health professionals and caregivers.

Evidence around the barriers and enablers associated with responsive feeding will also enable researchers to inform health professional communities and to develop and/or adapt any existing interventions. This has the potential to contribute to reduce inappropriate feeding and could be particularly important in the prevention of childhood obesity. It is anticipated that the findings may also inform intervention development in ensuring that barriers to responsive feeding are tackled. In regards to intervention development and improvement, it is important that where it is not possible to modify a particular determinant (for example, maternal executive functioning, or infant temperament) the intervention may be adapted to suit the caregivers specific needs.

### Potential limitations

This review will only include studies which are published in English, due to limitations in translation resources. This could mean excluding other relevant information based on language barriers. Secondly, unpublished literature will not be included, possibly leaning towards an increased risk of publication bias in the research that is included.

## Amendments

If we need to make any amendments to this protocol, we will give the date of each amendment, describe the change and provide rationale in this section.

## Data availability

### Underlying data

No data is associated with this article.

### Extended data

Figshare: CRiB Quantitative Data Extraction Form. https://doi.org/10.25411/aru.11498637.v1 ^60^

This project contains the following extended data:

- Quant Data Extraction.docx (Study data extraction form for quantitative data)

Figshare: CRiB Qualitative Data Extraction Form. https://doi.org/10.25411/aru.11498667.v1 ^53^

This project contains the following extended data:

- Qualitative Data Extraction Form Blank.xlsx (Study data extraction form for qualitative data)

### Reporting Guidelines

Repository: PRISMA-P checklist for ‘Barriers and enablers to Caregivers Responsive feeding Behaviour (CRiB): A mixed method systematic review protocol’. https://doi.org/10.25411/aru.11378844.v2 ^54^

Data are available under the terms of the Creative Commons Zero "No rights reserved" data waiver (CC0 1.0 Public domain dedication).
