# Supplementary material for: Barriers and enablers to Caregivers Responsive feeding Behaviour (CRiB): A mixed method systematic review protocol
Source: HRB Open Res. 2020 Jun 10;3:2. Originally published 2020 Jan 16. [Version 2] doi: 10.12688/hrbopenres.12980.2 (PMC7333359; doi:10.12688/hrbopenres.12980.2)
Supplement: Peer review history

# Peer review: Reviewer response for version 2

**DOI:** 10.21956/hrbopenres.14179.r27525

**Published:** 2020-07-13

**Authors:** Jackie Blissett

**Affiliations:** 1 Department of Psychology, School of Life and Health Sciences, Aston University, Birmingham, UK

The author have addressed my original suggestions well, I have no more comments to make.

Is the study design appropriate for the research question?

Yes

Is the rationale for, and objectives of, the study clearly described?

Yes

Are sufficient details of the methods provided to allow replication by others?

Yes

Are the datasets clearly presented in a useable and accessible format?

Not applicable

Reviewer Expertise:

Parent feeding practices, child temperament, infant and child feeding, childhood obesity.

I confirm that I have read this submission and believe that I have an appropriate level of expertise to confirm that it is of an acceptable scientific standard.

# Peer review: Reviewer response for version 2

**DOI:** 10.21956/hrbopenres.14179.r27526

**Published:** 2020-07-02

**Authors:** Heidi Bergmeier

**Affiliations:** 1 Monash Centre for Health Research and Implementation, Monash University, Melbourne, Australia

The authors have addressed all comments well.

Is the study design appropriate for the research question?

Yes

Is the rationale for, and objectives of, the study clearly described?

Yes

Are sufficient details of the methods provided to allow replication by others?

Yes

Are the datasets clearly presented in a useable and accessible format?

Not applicable

Reviewer Expertise:

Childhood obesity prevention, parent-child interactions, parent-child feeding interactions, child attachment, maternal and child obesity prevention in early parenting years, observational measures for assessing parent-child feeding interactions.

I confirm that I have read this submission and believe that I have an appropriate level of expertise to confirm that it is of an acceptable scientific standard.

# Peer review: Reviewer response for version 1

**DOI:** 10.21956/hrbopenres.14066.r27299

**Published:** 2020-05-07

**Authors:** Jackie Blissett

**Affiliations:** 1 Department of Psychology, School of Life and Health Sciences, Aston University, Birmingham, UK

This paper reports a mixed-methods systematic review protocol which aims to summarise and integrate current knowledge on the barriers and enablers of parental responsive feeding of infants and young children under two years. There is substantial evidence justifying the need for further investigation of early influences on children’s feeding, because these may be key predictors of later overweight and obesity. This is an important area of research, in need of consolidation of findings, and thus a systematic review of this literature will be a welcome contribution to the field. 

 The protocol is well designed and clearly justified. I have a few comments which I hope will fine tune the manuscript.

 Please consider adjusting phrasing to ensure ‘person first’ language and to avoid emphasis on individual personal responsibility: for example, ‘Children who are obese are more likely to be obese in adulthood,therefore exposing themselves to a higher risk of many chronic diseases’ would be better phrased as ‘Children who have obesity are more likely to have obesity in adulthood, which is associated with a higher risk of many chronic diseases’.

 A wide range of individual and social factors influencing parental use of responsive feeding are listed in the introduction but core literature is missing, which considers the role of the infant characteristics in responsive feeding. It is likely to be much easier to feed responsively when an infant has appetite within a healthy range, and when an infant gives clear hunger and satiety cues. Studies have shown that infant appetite is strongly related to later BMI and that this is in part genetically mediated (see Gemini cohort outputs). Parents also adjust their feeding in response to changes in weight or appetite across time (e.g. see studies on twins and siblings discordant for appetitive traits). Infants who are more temperamentally difficult may also demonstrate signals that are harder to ‘read’. These will also likely constitute important barriers/enablers to responsive feeding. It is of course necessary to focus the review on targets for behaviour change that are potentially modifiable, nonetheless, those parents who are feeding infants who are at the extremes of appetite or temperament traits may also be in greatest need of tailored intervention. Perhaps these factors can be considered in the proposed subgroup analysis, subject to the data yielded.

 I was curious why permissive or ‘laissez faire’ was not included in search terms related to feeding type concept, given that authoritarian and authoritative are included. 

 I am glad to see the mixed methods approach being taken in this review, this will yield a richer picture of the overall current state of knowledge. The application of the COM-B model to structure of the review will provide an excellent mapping for subsequent intervention planning.

Is the study design appropriate for the research question?

Yes

Is the rationale for, and objectives of, the study clearly described?

Yes

Are sufficient details of the methods provided to allow replication by others?

Yes

Are the datasets clearly presented in a useable and accessible format?

Not applicable

Reviewer Expertise:

Parent feeding practices, child temperament, infant and child feeding, childhood obesity.

I confirm that I have read this submission and believe that I have an appropriate level of expertise to confirm that it is of an acceptable scientific standard.

# Response

**Published:** 2020-06-03

**Authors:** Vicki Slater

**Affiliations:** Anglia Ruskin University, UK

Many thanks for taking time to review this protocol manuscript and for your helpful comments. We have spent time addressing these. Firstly, we went through the manuscript and the phrasing was changed in order to ensure ‘person first’ language to ensure there is no individual personal responsibility. Secondly, we have now included previous literature which includes the role of genetics (as in the Gemini outputs), and additionally the role of infant appetite, and appetitive traits. We have now also included literature on infant fussiness and how this relates to infant feeding. Finally, you mentioned why ‘permissive’ or ‘laissez faire were not included in our search terms. We decided that most often ‘laissez faire’ and ‘permissive’ are typically presented/written in combination with the term ‘feeding’, and as we included ‘feeding’ within our search terms, papers including ‘laissez faire’ or ‘permissive’ would therefore be picked up.

Once again, thank you for your comments and taking the time to review our manuscript.

# Peer review: Reviewer response for version 1

**DOI:** 10.21956/hrbopenres.14066.r27300

**Published:** 2020-04-27

**Authors:** Heidi Bergmeier

**Affiliations:** 1 Monash Centre for Health Research and Implementation, Monash University, Melbourne, Australia

Thank you for the opportunity to review the content of the published article titled: “ *Barriers and enablers to caregivers responsive feeding behaviour (CRiB): A mixed method systematic review protocol”.* I look forward to reading the results of the review once it has been finalised and published. With regards to the current protocol article, I have provided comments below relating to evidence included in the introduction, for the authors’ consideration, which I hope are helpful. 

**Introduction section:**

 Research and frameworks (including WHO Commission on Ending Childhood Obesity) would also suggest that the first 2000 days (conception to five years) are critical periods for child development, including establishing eating and weight gain patters. Why might the first two years be particularly important within this time frame? E.g., What specifically happens in a child’s life, in relation to eating/weight development, that makes it a critical stage? It seems important to mention that responsive feeding across the first two years will involve periods of breast/bottle feeding, transitioning to solids and for almost all infants, an exclusively solid diet - where the socialisation of children’s food preferences and eating behaviours and all manner of child reactions that go with it) are in full swing. Additionally, other critical foundations of child development are being laid during this early period (e.g., child attachment, self-regulation etc…) beyond the feeding scenario that may influence parent feeding/child eating-weight interactions. Hence we really need to consider child development (including weight gain) more holistically.

 As per the previous comment: The first sentence in second paragraph of this paper reminds us that feeding is a reciprocal process. Again in the discussion there is a very brief mention of child temperament. Yet the paper seems to focus mainly on the parent and what the parent does during feeding. It would seem appropriate to mention in the introduction some of the characteristics the child might bring to the ‘feeding table’, which need to be factored in when considering parent feeding. We also know that contributors to child overweight/obesity are more complex and broader than just parent feeding (e.g., genetics, intrauterine effects; social and health inequities, particularly now that we have evidence showing rates of under and over nutrition often go hand in hand in low to middle-income populations), and this should be acknowledged, even if beyond the scope of the proposed review.

 “ *Inability to recognize an infant’s weight is within a healthy weight range*…”: It would be helpful to clarify for the reader if this is in regards to infants being over or under weight or both and what parental feeding was observed according to types of weight perceptions. E.g., what did mothers do if they perceived their child to be underweight and then overweight?

*“These feeding styles have been associated with children developing unhealthy eating styles”*: Please confirm for the reader if this is referring to infants or children in older age groups and provide citations for specific studies  supporting the statement. Please use more recent citations if possible given that reference of review cited is from 2007. It also seems necessary to make it clear that evidence relating to child feeding and outcomes has been mixed (and there are reasons for this), but largely, it appears that responsive feeding may be protective.... 

 “ *Multiple factors may affect how caregivers engage with feeding behaviour…”:* It would be helpful to get a sense of the types of populations (socio-economic status, countries, ethnicity) that were investigated in the studies included in the review.

 Understanding the underlying factors that influence responsive feeding will contribute to the development of a caregiver-focused **supports** that **supports** responsive feeding: Could one of the “supports” used in this sentence be replaced by another word?

Is the study design appropriate for the research question?

Yes

Is the rationale for, and objectives of, the study clearly described?

Yes

Are sufficient details of the methods provided to allow replication by others?

Yes

Are the datasets clearly presented in a useable and accessible format?

Not applicable

Reviewer Expertise:

Childhood obesity prevention, parent-child interactions, parent-child feeding interactions, child attachment, maternal and child obesity prevention in early parenting years, observational measures for assessing parent-child feeding interactions.

I confirm that I have read this submission and believe that I have an appropriate level of expertise to confirm that it is of an acceptable scientific standard, however I have significant reservations, as outlined above.

# Response

**Published:** 2020-06-03

**Authors:** Vicki Slater

**Affiliations:** Anglia Ruskin University, UK

Thank you for taking the time to review our protocol manuscript, and for your insightful comments. We have taken the time to address these comments. To address your first comment, we have now included literature on why the first 2 years of life are particularly important, relating to feeding (such as breast/bottle feeding, the introduction of solid foods) but also to other developmental aspects (for example, attachment, and the development of self-regulation) which are occurring during this time. Secondly, we have now included literature on the role of infant characteristics (such as infant fussiness/temperament, infant appetite/appetitive traits) and other influences (such as genetics as in the Gemini outputs) on infant feeding. Throughout the manuscript we have addressed your comments regarding clarification; for example, a caregivers inability to recognise that their infant’s weight is within a heathy range, and the mixed evidence relating to child feeding. We have now specified the age range in which feeding styles have been associated with children developing unhealthy eating styles, with 2 additional more recent citations. In regards to your comment about the review cited, we have now included more information about this specific review, and the factors identified with relevant citations. Finally, we amended the repetition of the word ‘supports’.

Once again, thank you for your helpful comments and for taking the time to review this manuscript.
